# Supplementary material for: Fluctuating asymmetry and feather growth bars as biomarkers to assess the habitat quality of shade coffee farming for avian diversity conservation
Source: R Soc Open Sci. 2019 Aug 14;6(8):190013. doi: 10.1098/rsos.190013 (PMC6731696; doi:10.1098/rsos.190013)
Supplement: Test for normality(Shapiro Wilk test) and Kurtosis of signed FA in five bird species;Test for the significance of signed FA and directional asymmetry;Estimates of mean rectrix length for five bird species [file rsos190013supp1.docx]

**Supplementary materials**

Table S1. Tarsus length and rectrix mass signed FA analysis in five bird species. Species: Abyssinian Ground Thrush (AGT), African dusky Flycatcher (ADF), Grey-backed Camaroptera (GBC), Rüppell’s Robin-chat (RRC) and Tambourine Dove (TD). Natural=Natural forest and TFL=mean of the second outer most right and left rectrices.

| species | trait | Kurtosis | N | W | P |
| --- | --- | --- | --- | --- | --- |
| AGT | tarsus | 6.819 | 26 | 0.795 | 0.0001 |
|  | rectrix | 3.551 | 15 | 0.862 | 0.0257 |
| ADF | tarsus | 3.08 | 43 | 0.889 | 0.0006 |
|  | rectrix | 15.509 | 20 | 0.3705 | <0.0001 |
| GBC | tarsus | 3.08 | 28 | 0.902 | 0.0128 |
|  | rectrix | 11.231 | 15 | 0.5852 | <0.0001 |
| RRC | tarsus | 2.534 | 125 | 0.866 | <0.0001 |
|  | rectrix | 16.929 | 96 | 0.7683 | <0.0001 |
| TD | tarsus | 0.333 | 97 | 0.937 | <0.937 |
|  | rectrix | 24.749 | 77 | 0.4964 | <0.001 |

Table S2. Estimates of bilateral traits asymmetry for tarsus length and rectrix mass. Level of fluctuating asymmetry (FA) and directional asymmetry (DA) relative to measurement error (ME) were obtained from mixed regression model analysis and as a basis to calculate repeatability [intra-correlation coefficient(ICC) = FA/(FA+ME)]. Measured trait (tarsus length or rectrix mass) values included in the models a response variable, side both as fixed and random effects and individual Id as a subject.

| Trait | FA | ME | FA (LR-test) | ICC% | DA (F-test) | p |
| --- | --- | --- | --- | --- | --- | --- |
| Tarsus | 0.0513 | 0.179 | χ^2^ = 789.7, df=1, P = 0 | 23.3% | F_1,342_ = 12.27 | 0.0005 |
| Rectrix | 1.2 x 10^-5^ | 0.4404 | χ^2^ = 449.1, df=1, P = 0 | 2.7 x 10^-5^ | F_1,238_ = 2.78 | 0.0967 |

Table S3. Estimates of mean rectrix length from general linear mixed models, where mean rectrices lengths (mean of the second outer most right and left) as response variable, forest type as fixed effect, and sampling location as random effect. Species: Abyssinian Ground Thrush (AGT), African dusky Flycatcher (ADF), Grey-backed Camaroptera (GBC), Rüppell’s Robin-chat (RRC) and Tambourine Dove (TD). Natural=Natural forest.

| species | effect | estimate | SE | df | t | P |
| --- | --- | --- | --- | --- | --- | --- |
| AGT | Intercept | 85.80 | 2.23 | 14 | 38.68 | <0.0001 |
|  | Forest type (natural) | 0.48 | 2.68 | 14 | -0.18 | 0.8597 |
| ADF | Intercept | 50.19 | 0.629 | 19 | 79.81 | <0.0001 |
|  | Forest type (natural) | -0.87 | 0.8264 | 19 | -1.05 | 0.3061 |
| GBC | Intercept | 38.72 | 0.8765 | 13 | 44.18 | <0.0001 |
|  | Forest type (natural) | 0.44 | 1.39 | 13 | 0.32 | 0.7533 |
| RRC | Intercept | 64.05 | 0.51 | 5.75 | 125.04 | <0.0001 |
|  | Forest type (natural) | -0.31 | 0.91 | 12 | -0.34 | 0.740 |
| TD | Intercept | 20.63 | 0.68 | 77 | 1.42 | .1587 |
|  | Forest type (natural) | 0.98 | 0.69 | 77 | 1.42 | 0.1587 |
